# Single-Center Retrospective Subgroup Analysis of “Primary Aortic” (Aneurysm, Aortic Dissection, PAU) and “Secondary Aortic” (Iatrogenic, Trauma, Aortoesophageal Fistula) Indications for Emergency TEVAR

**DOI:** 10.3390/jcm12124037

**Published:** 2023-06-13

**Authors:** Artur Rebelo, Jumber Partsakhashvili, Ulrich Ronellenfitsch, Endres John, Jörg Kleeff, Jörg Ukkat

**Affiliations:** Department of General, Abdominal, Vascular and Endocrine Surgery, University Hospital Halle (Saale), Martin-Luther-University Halle-Wittenberg, 06120 Halle, Germany; jumber.partsakhashvili@uk-halle.de (J.P.); ulrich.ronellenfitsch@uk-halle.de (U.R.); endres.john@uk-halle.de (E.J.); joerg.kleeff@uk-halle.de (J.K.); joerg.ukkat@uk-halle.de (J.U.)

**Keywords:** TEVAR, aortic emergencies, aortoesophageal fistula, trauma, iatrogenic, aortic aneurysm, aortic dissection

## Abstract

Background: The aim of this study was to analyze the outcome of emergency thoracic endovascular aortic repair (TEVAR) in the treatment of “primary aortic” (aneurysm, aortic dissection, penetrating aortic ulcer (PAU)) and “secondary aortic” (iatrogenic, trauma, and aortoesophageal fistula) pathologies. Methods: Retrospective review of a cohort of patients treated at a single tertiary referral center from 2015 to 2021. The primary end point was postoperative in-hospital mortality. Secondary end points were the duration of the procedure, duration of postoperative intensive care treatment, length of hospital stay, and the nature and severity of postoperative complications according to the Dindo–Clavien classification. Results: A total of 34 patients underwent TEVAR for emergency indications. Twenty-two patients were treated for primary and twelve patients for secondary aortic pathologies. Concerning in-hospital mortality, no statistically significant difference could be observed between the primary and secondary aortic groups (27.3% vs. 33.3%, *p* = 0.711). Patients with an aortoesophageal fistula had a mortality rate of 66.7%. Postoperative morbidity (Dindo–Clavien > 3) was also not statistically significantly different between the primary and secondary aortic groups (36.4% vs. 33.3%, *p* = 0.86). Preoperative hemoglobin level (*p* < 0.001 for mortality, *p* = 0.002 for morbidity), hemoglobin level difference (*p* = 0.022, *p* = 0.032), postoperative creatinine level (*p* = 0.009, *p* = 0.035), and pre- and postoperative lactate levels (*p* < 0.001 for both mortality and morbidity) were found to be independent factors associated with postoperative mortality and morbidity (Dindo–Clavien > 3), respectively. The preoperative creatinine level was found to be associated with mortality (*p* = 0.024) but not morbidity. Conclusions: Morbidity and in-hospital mortality are still considerable after emergency TEVAR for both primary and secondary aortic indications. Pre- and postoperative levels of hemoglobin, creatinine, and lactate may be valuable to predict patient outcomes.

## 1. Introduction

In 1987 in Ukraine, Volodos et al. performed the first thoracic endovascular aortic repair (TEVAR) to treat a traumatic thoracic aortic aneurysm. Later, Parodi et al. and Dake et al. reported on successful TEVAR procedures [1,2,3]. Thoracic aortic emergencies involve several etiologies, including rupture of thoracic aneurysms, complicated acute Stanford type B dissections, penetrating aortic ulcers (PAU), injury, and iatrogenic, aortoesophageal, and aortobronchial fistulas [4]. 

With the improvement of endovascular stent grafts and growing expertise in endovascular surgery, emergency surgery for pathologies of the thoracic aorta is nowadays dominated by TEVAR procedures. 

In particular, the use of endografts in traumatic aortic rupture was a revolution in the treatment of these patients, with large multicentric studies showing a clear advantage over open surgery in terms of in-hospital mortality [5,6,7]. Despite the absence of RCTs comparing open and endovascular surgery, TEVAR is nowadays considered the gold standard for treating traumatic thoracic aortic lesions [8,9,10]. Lower in-hospital mortality rates for TEVAR over open surgery were also observed regarding ruptured thoracic aortic aneurysms and aortic dissections [11,12,13,14,15,16,17,18]. Regarding complicated type B aortic dissections, the evidence is scarce, and no RCTs have been published comparing open with endovascular therapy [19]. A meta-analysis from 2021 compared TEVAR for complicated and uncomplicated aortic dissections. No differences in in-hospital mortality (OR 0.01 (0–0.05), *p* = 0.007) were observed between the groups [20].

Aortoesophageal fistula is a rare and frequently fatal condition, even with early diagnosis and treatment. A primary aortoesophageal fistula arises from thoracic aortic aneurysms, presence of a foreign body, or tumor. Secondary fistulas are related to endovascular or esophagectomy procedures [21]. TEVAR also presents advantages compared to open repair in terms of mortality and morbidity, despite only data from small cohort studies being available [22,23,24,25,26,27,28,29,30,31]. Some authors also suggest the use of TEVAR as a bridging procedure in the treatment of locally advanced esophageal cancer with aortic affection to facilitate chemoradiotherapy and, eventually, subsequent resection [32,33]. Lastly, case reports and small case series reported on the feasibility of TEVAR for radiogenic and iatrogenic lesions of the aorta [17,30,34]. 

The aim of this study was to analyze the outcome of endovascular interventions in the emergency treatment of thoracic aortic pathology in a single tertiary referral centre in Germany, comparing “primary aortic” (aneurysm, aortic dissection, PAU) with “secondary aortic” (iatrogenic, trauma, and aortoesophageal fistula) lesions.

## 2. Methods

All patients 18 years and older at the time of surgery who were treated for an aortoesophageal fistula, trauma, iatrogenic thoracic aortic lesions, and thoracic aortic aneurysms, dissections, or PAU and who underwent endovascular treatment in an emergency setting at the Department for Visceral, Vascular, and Endocrine Surgery at the University Hospital Halle (Saale), Germany from 2015 to 2021 were included in the study. 

The primary outcome of the study was postoperative in-hospital mortality. Secondary outcomes were the duration of the procedure, duration of postoperative intensive care treatment, length of hospital stay, and the nature and severity of postoperative complications according to the Dindo–Clavien classification [35]. All outcomes and patients’ demographic characteristics and co-morbidities (hypertension, diabetes mellitus, chronic heart disease, renal insufficiency, and chronic obstructive pulmonary disease, all defined as patients having chronic medication) were collected by retrospective chart review and entered into a study database following anonymization. As all data were processed fully anonymously, the need for ethical approval and informed consent of patients was waived by the competent ethical committee (Ethics Committee of the Medical Faculty of the Martin-Luther-University Halle-Wittenberg) according to Section 17 of the Hospital Act of the Federal State of Saxony-Anhalt and Section 15 of the Saxony-Anhalt Medical Association’s professional code of conduct. Pearson’s X^2^ test was used to identify independent factors associated with early death and postoperative morbidity. The Mann–Whitney test was used to compare continuous and ordinal variables, and the Chi-square test was used to compare categorical variables. A *p*-value of 0.05 determined statistical significance. IBM SPSS Statistics 27 (IBM, Chicago, IL, USA) was used to perform the analysis. 

## 3. Results

### 3.1. Demographics and Clinical Characteristics 

A total of 34 patients, 22 with primary aortic and 12 with secondary aortic indications, underwent emergency TEVAR during the study period. In the “primary aortic” (aneurysm, aortic dissection, PAU) and “secondary aortic” (iatrogenic, trauma, aortoesophageal fistula) groups, 72.7% and 83.3% were male, respectively (*p* = 0.486). The mean age was 71.4 and 51.9 years (*p* = 0.002) in the primary aortic and secondary aortic groups, respectively. Patients in the aortic group had a higher prevalence of high blood pressure (77.2% vs. 66.7%, *p* = 0.003), diabetes mellitus (13.6% vs. 0%, *p* = 0.18), chronic heart disease (27.3% vs. 16.7%, *p* = 0.49), hyperlipidemia (13.6% vs. 0%, *p* = 0.18), renal insufficiency (27.3% vs. 0%, *p* = 0.046), and COPD (18.2 vs. 0%, *p* = 0.116). A summary of relevant demographics and co-morbidities is presented in Table 1. 

### 3.2. Etiology, Classification, Laboratory Values and Outcomes

**Figure 1:**
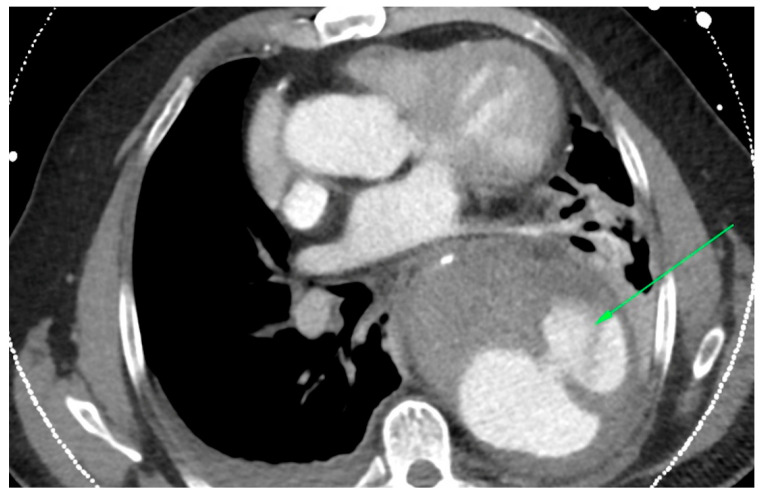
CT-scan of a thoracic aortic aneurysm rupture (green arrow).

**Figure 2:**
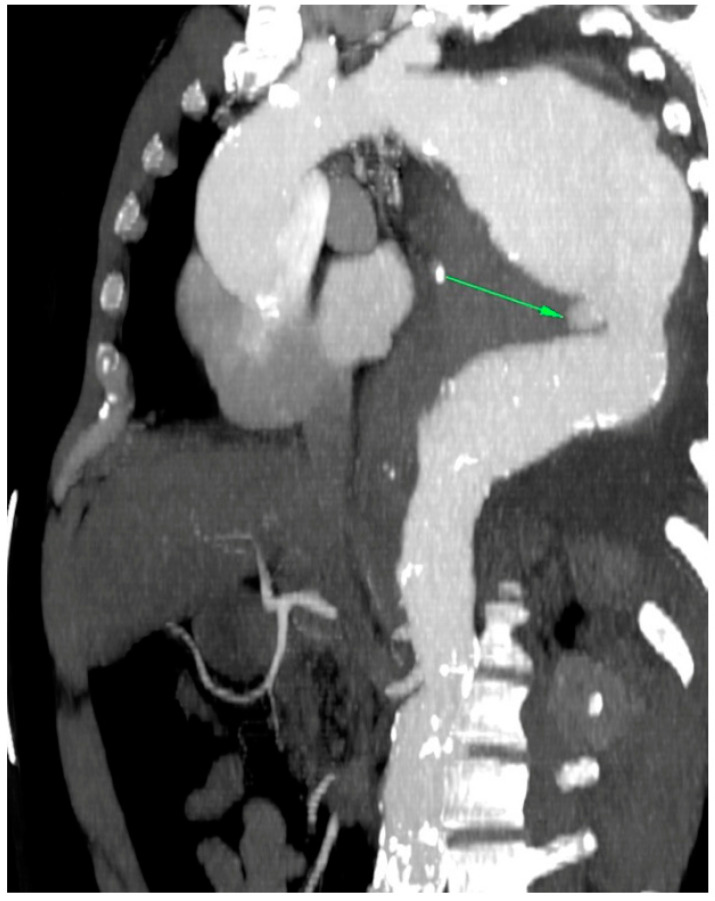
CT-scan of a thoracic aortic aneurysm rupture with an aortoesophageal fistula (green arrow).

**Figure 3:**
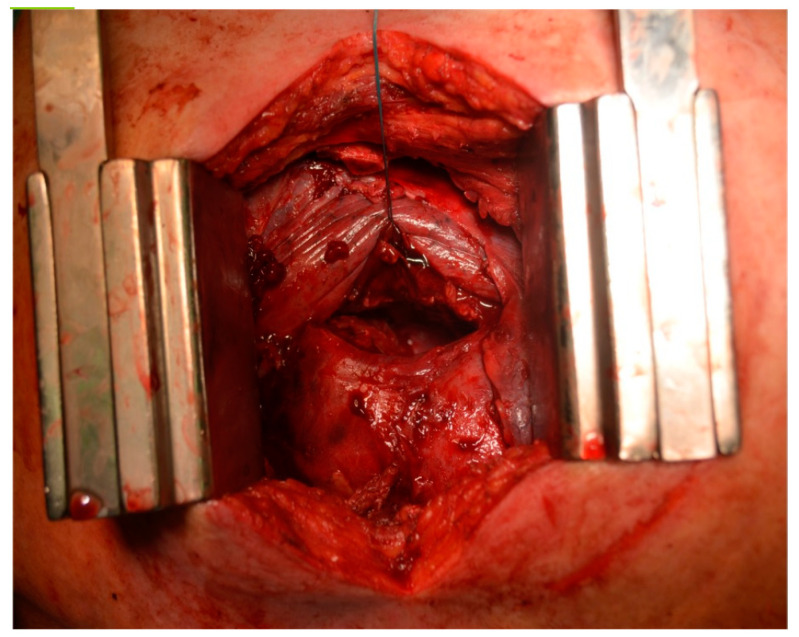
Intraoperative image of an aortoesophageal fistula.

**Figure 4:**
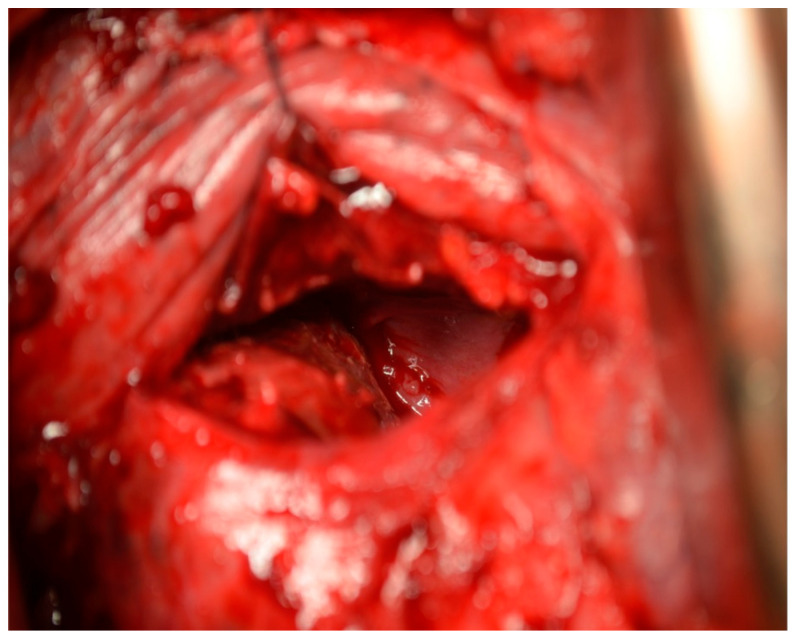
Intraoperative image of an aortoesophageal fistula.

In the primary aortic group, 64% had an aneurysm (Figure 1), 18% had an aortic dissection, and 18% a PAU. In the secondary aortic group, 17% had an iatrogenic aortic lesion after spine surgery, 58% a trauma, and 25% an aortoesophageal fistula (Figure 2 and Figure 3). All patients underwent CT scan for diagnosis and after the surgery (Figure 4). Percutaneous access was performed in 31.8% of the patients in the primary aortic and 41.7% in the secondary aortic group (*p* = 0.566). An operation in local anesthesia was performed in 31.8% of the patients in the primary aortic group and 25% in the secondary aortic group (*p* = 0.677). Median duration of hospital stay was longer in the primary aortic group compared to the secondary aortic group (14.5 vs. 8 days, *p* = 0.746). Simultaneous surgery was similarly frequent in both groups (22.7% vs. 25%, *p* = 0.881). Procedures included: hepatic artery bypass, aortic arch debranching, surgical treatment of a pelvic fracture, thoracotomy, bowel resection, trepanation, ECMO implantation, and splenectomy. 

Regarding the preoperative laboratory values, anemia and elevated lactate and creatinine levels were more frequent in the secondary aortic group when compared to the primary aortic group (Hb 6.36 (±1.1) vs. 6.62 (±1.1) mmol/L, *p* = 0.942, 92.1 (±43.6) vs. 80.46 (±29.1) mmol/L, *p* = 0.528, and 5.2 (±5.57) vs. 1.35 (±0.21) umol/L, *p* = 0.029). Regarding the postoperative laboratory values, anemia and elevated lactate and creatinine levels were also more frequent in the secondary aortic group when compared to the primary aortic group (Hb 5.93 (±0.9) vs. 5.65 (±0.071) mmol/L, *p* = 0.9, 97.2 (±47.8) vs. 44.5 (±20.5), *p* = 0.614, and 4.1 (±4.7) vs. 1.15 (±1.43) umol/L, *p* = 0.233). 

Concerning in-hospital mortality, no statistically significant difference could be observed between the primary and secondary aortic groups (27.3% vs. 33.3%, *p* = 0.711). No patient died after treatment for PAU or iatrogenic lesions. Mortality rates after TEVAR for aneurysm, dissection and traumatic lesions were 35.7%, 25% and 28.6%, respectively. Patients with aortoesophageal fistula had the highest mortality rate (66.7%). Morbidity (Dindo–Clavien grade > 3) was not significantly different between the primary and secondary aortic groups (36.4% vs. 33.3%, *p* = 0.86). Morbidity included groin hematoma, thoracic bleeding, and multiple organ dysfunction. Two patients underwent a second TEVAR procedure for endoleak type I during the hospital stay. A summary is presented in Table 2. 

### 3.3. Independent Factors Associated with Early Death and Postoperative Morbidity

Preoperative hemoglobin level (*p* < 0.001 for mortality and *p* = 0.002 for morbidity), hemoglobin level difference (*p* = 0.022 and *p* = 0.032), postoperative creatinine level (*p* = 0.009 and *p* = 0.035), and pre- and postoperative lactate levels (*p* < 0.001 for both mortality and morbidity) were found to be independent factors associated with postoperative mortality and morbidity (Dindo–Clavien > 3), respectively. Preoperative creatinine level was found to be associated with mortality (*p* = 0.024) but not morbidity. A summary is presented in Table 3. 

## 4. Discussion 

In this retrospective study, we report our single center experience with emergency TEVAR for primary and secondary aortic lesions. Despite patients with secondary aortic pathology being younger and having fewer co-morbidities, no statistically significant difference could be observed concerning mortality and morbidity when compared to the primary aortic group. This could be related to the more unstable condition of these patients, mirrored by higher lactate levels. In other studies, patients with primary aortic pathologies such as aneurysms were also found to have more co-morbidities [36]. Our data are comparable to a recent multicentric VASCUNET register study from 11 countries involving 9,518 TEVAR procedures for thoracic aortic aneurysms, type B aortic dissections, and traumatic aortic injuries. After repair for ruptured thoracic aneurysm, perioperative mortality was 26.8% [37]. Data from centers in the UK and Germany concerning emergency repair for thoracic aortic aneurysms also showed similar death rates of 30% and 44%, respectively [38,39]. 

Concerning traumatic aortic injuries, our small patient collective (n = 7) had a mortality rate of 26.8%. We did not include patients undergoing delayed repair, which may explain this relatively high mortality rate. In a retrospective review of a US National Trauma Database, 2821 patients with blunt traumatic aortic injury were included. Mortality was higher in patients undergoing early TEVAR compared to delayed TEVAR across injury severity groups and was independent of serious extra-thoracic injuries (9.8% vs. 4.4%, *p* = 0.001) [40]. 

**Figure 5:**
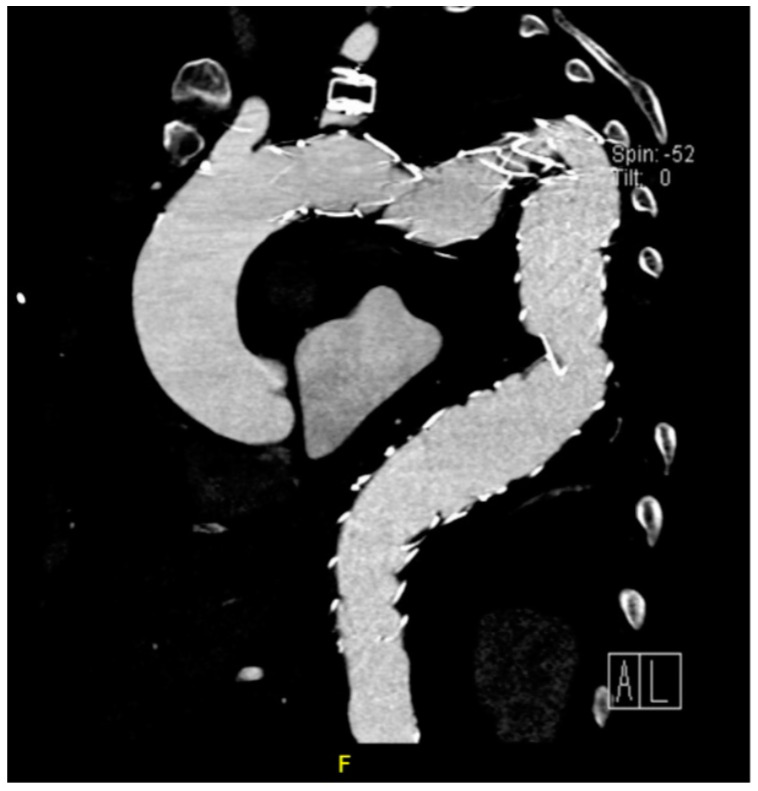
Postoperative CT scan after TEVAR.

In the patients included in our analysis, there were three patients treated for an aortoesophageal fistula. Only one patient, treated for primary fistula with esophagectomy and TEVAR in the same procedure, survived (Figure 2, Figure 3, Figure 4 and Figure 5). This patient is still alive and in follow-up. The other two patients treated for a secondary fistula due to anastomotic leak after esophagectomy for esophageal cancer died after TEVAR. The incidence of an aortoesophageal fistula after TEVAR for primary aortic pathologies is low, as demonstrated in the European Registry of Endovascular Aortic Repair Complications (1.5%). In this cohort, the highest 1-year survival (46%) could be achieved via an aggressive treatment, including radical esophagectomy and aortic graft replacement [41]. In our patient population, we did not treat patients with a secondary fistula after TEVAR. In a 2014 review, 55 articles were included which reported on 72 patients treated with TEVAR for aortoesophageal fistula. Similar to our data (100% technical success rate), the technical success rate was 87.3%. Nevertheless, the overall 30-day mortality was significantly lower (19.4%) than in our series [42]. In a 2009 meta-analysis, 43 patients with aortoesophageal fistula were identified. Mortality after TEVAR was 19%. Patients who underwent esophageal surgery in the first month after TEVAR had lower fistula-related mortality during 6 months of follow-up compared to the other patients (*p* = 0.018) [43].

In our patient collective, we did not perform routine simultaneous hemothorax decompression. In a small case series of 17 patients, lower rates of respiratory failure (50.0% vs. 16.7%, *p* = 0.198) and 90-day mortality (62.5% vs. 33.3%, *p* = 0.280) were observed for patients with immediate hemothorax decompression [44]. We opted to perform a CT scan and then perform a staged decompression after surgery if needed. 

The second finding is related to the independent predictors of mortality and morbidity identified in our patient population: preoperative hemoglobin level, hemoglobin level difference (*p* = 0.022), postoperative creatinine level, and pre- and postoperative lactate levels, confirming the importance of the perioperative blood, fluid, and electrolyte management of these patients. These results must be carefully interpreted as we could not assess data regarding transfusion of blood products. How far these findings are relevant in clinical practice should be assessed in future prospective studies. Developing a risk score may be helpful in defining the prognosis of these patients. 

This study has some limitations. The main drawback is that it is based on a small number of patients. Its retrospective design is another significant limitation, increasing the risk of bias considerably. It was not possible to collect data on operation timing and blood transfusion, for example. Therefore, the data should be carefully interpreted and applied. Nevertheless, the findings of this work may provide useful information for clinicians treating thoracic aortic emergencies. 

Thoracic aortic emergencies are a challenge for vascular surgeons. In a small patient collective, we showed that endovascular techniques were feasible with acceptable morbidity and in-hospital mortality for pathologies which a priori have a limited prognosis. Early referral to vascular and surgical oncology centers with expertise in thoracic endovascular treatments and esophageal surgery may be critical for these patients. Multidisciplinary teams are needed for the treatment of these complex pathologies. In order to more thoroughly assess, and to possibly improve, outcomes of these procedures, multicentric trials and international databases and clinical registries seem desirable. Such larger-scale research endeavors providing real-world data on these procedures may paint a clearer picture of relevant outcomes and how we may be able to positively influence them.

## Figures and Tables

**Table 1:** Summary of the baseline and clinicopathologic features in 34 patients undergoing emergency TEVAR from 2015–2021 (Mann–Whitney test for continuous and ordinal variables and Chi-square test to the categorical variables used to compared primary and secondary Aortic groups).

Variable	Primary Aortic				Secondary Aortic				*p* Value
	Aneurysm (n = 14)	Dissection (n = 4)	PAU (n = 4)	Total (n = 22)	Iatrogenic (n = 2)	Trauma (n = 7)	Aortoesophageal Fistula (n = 3)	Total (n = 12)	
Male gender (%)	64.3	75	100	72.7	100	71.4	100	83.3	0.486
**Age (years) mean (SD)**	**73.2 (12.3)**	**69.75 (13.2)**	**66.5 (12.6)**	**71.4**	**67 (1.4)**	**44.1 (17.5)**	**60 (6)**	**51.9**	**0.002**
**High blood pressure (%)**	**85.7**	**50**	**75**	**77.2**	**50**	**0**	**66.7**	**25**	**0.003**
DM (%)	7.1	0	50	13.6	0	0	0	0	0.18
CHD (%)	28.5	25	25	27.3	50	0	33.3	16.7	0.486
Hyperlipidemia (%)	21.4	0	0	13.6	0	0	0	0	0.18
**Renal insufficiency (%)**	**28.6**	**0**	**50**	**27.3**	**0**	**0**	**0**	**0**	**0.046**
COPD (%)	21.4	25	0	18.2	0	0	0	0	0.116

CHD: coronary heart disease; DM: diabetes mellitus; COPD: chronic obstructive pulmonary disease.

**Table 2:** Preoperative laboratory values, technical details, and postoperative outcomes in 34 patients undergoing emergency TEVAR from 2015–2021 (Mann–Whitney test for continuous and ordinal variables and Chi-square test to the categorical variables used to compared primary and secondary aortic groups).

Variable	Primary Aortic				Secondary Aortic				*p* Value
	Aneurysm (n = 14)	Dissection (n = 4)	PAU (n = 4)	Total (n = 22)	Iatrogenic (n = 2)	Trauma (n = 7)	Aortoesophageal Fistula (n = 3)	Total (n = 12)	
Percutaneous (%)	42.85	25	0	31.8	0	43	66.7	41.7	0.566
Hospital stays (days) Median (SD)	14 (10.8)	15.5 (3.5)	18 (13.9)	14.5 (10.1)	111.5 (153.4)	5 (8.7)	96 (33.55)	8 (72.7)	0.746
Local anesthesia (%)	14.3	75	50	31.8	0	28.6	33.3	25	0.677
Hemoglobin level preoperative (mmol/L) Mean (SD)	6.5 (1.3)	6.98 (0.27)	6.65 (1.12)	6.62 (1.1)	6.7 (1.4)	6.47 (1.45)	5.867 (1.8)	6.36 (1.1)	0.942
Hemoglobin level postoperative (mmol/L) Mean (SD)	5.99 (0.98)	5.88 (0.70)	6.3 (1.11)	6.01 (0.93)	5.65 (0.071)	6.014 (1.232)	5.9 (1.71)	5.93 (1.12)	0.9
Creatinine level preoperative (µmol/L) Mean (SD)	80.43 (32.2)	85.25 (36.6)	75.75 (7.1)	80.46 (29.1)	59.5 (28.99)	95 (46.4)	107 (46.5)	92.1 (43.6)	0.528
Creatinine level postoperative (µmol/L) Mean (SD)	87.7 (35.9)	76.5 (26.4)	83 (11.5)	84.8 (30.5)	44.5 (20.5)	97.86 (44.39)	130.68 (44.8)	97.2 (47.8)	0.614
**Lactate level preoperative (mmol/L) Mean (SD)**	**1.54 (1.38)**	**2.25 (2.13)**	**0.775 (0.15)**	**1.53 (1.429)**	**1.35 (0.21)**	**5.6 (6.16)**	**6.833 (6.145)**	**5.2 (5.57)**	**0.029**
Lactate level postoperative (mmol/L) Mean (SD)	1.69 (1.6)	1.78 (1.38)	0.73 (0.34)	1.53 (1.43)	1.15 (0.35)	3.96 (4.89)	6.233 (5.67)	4.1 (4.7)	0.233
Simultaneous surgery (%)	21.4	50	0	22.7	0	28.6	33.3	25	0.881
In-Hospital mortality (%)	35.7	25	0	27.3	0	28.6	66.7	33.3	0.711
Dindo-Clavien ≥ 3 (%)	42.9	25	25	36.4	0	28.6	66.7	33.3	0.86
Surgery duration (min)	102.6 (84.3)	118.75 (86.5)	42.5 (12.1)	94.6 (78.5)	109.5 (96.9)	58 (26.4)	87.3 (63.9)	73.9 (49.2)	0.493

**Table 3:** Pearson’s X^2^ test analysis of independent factors associated with in-hospital mortality and postoperative morbidity (Dindo–Clavien ≥ 3) in the study population.

Mortality	Yes (n = 10)	No (n = 24)	*p* Value
Etiology (non-aortic) (%)	40	33.3	0.721
Male gender (%)	70	79.2	0.58
Age (years) (Mean SD)	64.4 (19.7)	64.5 (15.5)	0.98
High blood pressure (%)	50	62.5	0.515
DM (%)	0	12.5	0.255
CHD (%)	10	29.2	0.243
Hyperlipidemia (%)	10	8.3	0.881
Renal insufficiency (%)	16.7	20	0.823
COPD (%)	10	12.5	0.843
Percutaneous (%)	30	37.5	0.688
**Hemoglobin level preoperative (mmol/L) (Mean SD)**	**5.5 (1.09)**	**6.95 (0.991)**	**<0.001**
Hemoglobin level postoperative (mmol/L) (Mean SD)	5.68 (1.91)	6.121 (0.911)	0.249
**Hemoglobin level difference (preoperative–postoperative) (mmol/L) (Mean SD)**	**−0.18 (1.072)**	**0.833 (1.14)**	**0.022**
**Creatinine level preoperative (µmol/L) (Mean SD)**	**105 (39.9)**	**76 (29.1)**	**0.024**
**Creatinine level postoperative (µmol/L) (Mean SD)**	**114.4 (43.4)**	**76 (29.1)**	**0.009**
Creatinine level difference (preoperative–postoperative) (mmol/L) (Mean SD)	−9.4 (28.336)	−2.625 (17.093)	0.395
**Lactate level preoperative (mmol/L) (Mean SD)**	**6.38 (5.45)**	**1.35 (1.21)**	**<0.001**
**Lactate level postoperative (mmol/L) (Mean SD)**	**5.78 (4.297)**	**1.025 (0.481)**	**<0.001**
Lactate level difference (preoperative–postoperative) (mmol/L) (Mean SD)	0.321 (1.101)	−9.4 (28.336)	0.625
Simultaneous surgery (%)	40	16.7	0.153
Surgery duration (min) (Mean SD)	111.8 (94.7)	77.1 (55.2)	0.189
**Morbidity** **(Dindo–Clavien > 3) (%)**	**100**	**8.3**	**<0.001**
Local anesthesia (%)	30	29	0.963
Hospital stay (days) (Mean SD)	27.7 (36.6)	29.5 (50.1)	0.917
**Morbidity** **(Dindo–Clavien ≥ 3)**	**Yes (n = 12)**	**No (n = 22)**	***p*** **Value**
Etiology (non-aortic) (%)	33.3	36.4	0.865
Male gender (%)	75	77.3	0.886
Age (years) (Mean SD)	66.8 (18.8)	63.2 (15.5)	0.552
High blood pressure (%)	58.3	59.1	0.967
DM (%)	0	13.6	0.191
CHD (%)	16.7	27.3	0.501
Hyperlipidemia (%)	16.7	4.5	0.247
Renal insufficiency (%)	25	13.6	0.422
COPD (%)	16.7	9.1	0.527
Percutaneous (%)	25	40.9	0.369
**Hemoglobin level preoperative (mmol/L) (Mean SD)**	**5.69 (1.21)**	**6.98 (0.96)**	**0.002**
Hemoglobin level postoperative (mmol/L) (Mean SD)	5.7 (1.1)	6.15 (0.94)	0.217
**Hemoglobin level difference (preoperative–postoperative) (mmol/L) (Mean SD)**	**−0.008 (1.263)**	**0.832 (1.079)**	**0.032**
Creatinine level preoperative (µmol/L) (Mean SD)	99.75 (38.2)	76.3 (30.5)	0.058
**Creatinine level postoperative (µmol/L) (Mean SD)**	**107.25 (42.7)**	**79.3 (30.7)**	**0.035**
Creatinine level difference (preoperative–postoperative) (mmol/L) (Mean SD)	−7.5 (26.012)	−3.045 (17.826)	0.529
**Lactate level preoperative (mmol/L) (Mean SD)**	**5.48 (5.445)**	**1.38 (1.25)**	**0.002**
**Lactate level postoperative (mmol/L) (Mean SD)**	**4.98 (4.3)**	**1.03 (0.495)**	**<0.001**
Lactate level difference (preoperative–postoperative) (mmol/L) (Mean SD)	0.492 (2.026)	0.355 (1.145)	0.684
Simultaneous surgery (%)	41.7	13.6	0.069
Surgery duration (min) (Mean SD)	116.5 (95.4)	71.4 (45.6)	0.07
**Mortality (%)**	**83**	**0**	**<0.001**
Local Anesthesia (%)	33.3	27.3	0.721
Hospital stay (days) (Mean SD)	28.18 (33.2)	29.46 (52.39)	0.939

CHD: coronary heart disease; DM: diabetes mellitus; COPD: chronic obstructive pulmonary disease.

## Data Availability

The data that support the findings of this study are available from the corresponding author, but restrictions apply to the availability of these data, which were used under license for the current study and so are not publicly available. Data are, however, available from the corresponding author upon reasonable request and with permission of the ethics committee of the University Hospital Halle (Saale).
